# 1,2,3,6-Tetra-O-Galloyl-β-D-Glucopyranose Induces Apoptosis and Ferroptosis in Colon Cancer Cells by Inhibiting the Wnt/β-Catenin Signaling Pathway

**DOI:** 10.4014/jmb.2503.03050

**Published:** 2025-07-18

**Authors:** Suhyeon Kim, MinKyun Na, Sangtaek Oh

**Affiliations:** 1 Department of Bio and Fermentation Convergence Technology, Kookmin University, Seoul 02707, Republic of Korea; 2 College of Pharmacy, Chungnam National University, Daejeon 34134, Republic of Korea

**Keywords:** β-Catenin, 1,2,3,6-tetra-O-galloyl-β-D-glucopyranose, phosphorylation, degradation, colon cancer

## Abstract

Molecular irregularities in the canonical Wnt pathway that lead to the stabilization of β-catenin are common in colon cancer. Here, we identified 1,2,3,6-Tetra-O-galloyl-β-D-glucopyranose (TAGP), separated from *Trapa japonica*, as an inhibitor of canonical Wnt signaling. TAGP facilitated the phosphorylation of Ser33/37 and the degradation of β-catenin, which had accumulated due to Wnt3a-conditioned medium or the inhibitor 6-bromoindirubin-3’-oxime that targets glycogen synthase kinase-3β (GSK-3β). Additionally, TAGP lowered the levels of Cyclin D1 and c-Myc, which are regulated by β-catenin/T-cell factor (TCF) and showed antiproliferative activity in colon cancer cells. Furthermore, TAGP triggered apoptosis, as demonstrated by the activation of caspases 3 and 7, in conjunction with raising the number of Annexin-V-positive cells. It also promoted ferroptosis, as shown by the buildup of lipid peroxides and Fe^2+^ in the cells. Taken together, TAGP enhances β-catenin turnover, indicating its potential as a chemotherapeutics for colon cancer in humans.

## Introduction

Colorectal cancer (CRC) ranks as the second leading cause of cancer deaths worldwide and frequently affects the human digestive system [1]. It is closely linked with upregulation of canonical Wnt signaling [2]. Alterations acquired in the adenomatous polyposis coli (APC) gene are currently acknowledged as the first genetic changes identified in the onset of sporadic colorectal cancer [3]. Moreover, patients with colorectal cancer have been found to have changes in the N-terminal amino acid residues of β-catenin [4]. These mutations cause an excessive buildup of cytosolic β-catenin, allowing it to enter the nucleus and trigger the expression of target genes, which is crucial in colorectal tumorigenesis [5, 6]. Therefore, accelerating β-catenin turnover represents a possible therapeutic approach for preventing and treating colon cancer.

*Trapa japonica* is an annual water plant that belongs to the Trapaceae family [7]. This medicinal plant is prevalent in lakes and waterways worldwide, including regions such as Korea, China, India, and North America [8]. It has been utilized for culinary and medicinal purposes, including its antioxidative and antidiabetic properties and lowering blood glucose levels [9, 10]. In addition, Extracts from *Trapa japonica* demonstrate anti-proliferative effects on various cancer cell types [11]. Nonetheless, the anti-proliferative mechanism and bioactive compounds have yet to be explored in colon cancer cells. This study found that TAGP is a significant compound driving cytotoxic effects against colon cancer cells. TAGP induces both ferroptosis and apoptosis in colon cancer cells by enhancing the breakdown of pathogenic β-catenin.

## Methods and Materials

### Cell Culture, Reporter Assays, and Chemicals

HEK293, SW480, HCT116 and Wnt3a-secreting L cells were sourced from the American Type Culture Collection (USA) and cultured in DMEM containing 10% fetal bovine serum (FBS) and 1% penicillin/streptomycin. Dual Luciferase Assay Kits (Promega, USA) and the Phospha-Light Assay Kit (Applied Biosystems, USA) were used to measure β-catenin-dependent firefly luciferase and secreted alkaline phosphatase activities, respectively, according to the manufacturer’s guidelines. 6-Bromoindirubin-3’-oxime (BIO) and MG-132 were purchased from Sigma-Aldrich (USA).

### Western Blot Analysis

Cytoplasmic and whole-cell proteins were isolated using the method described in [12]. We conducted Western blot analysis according to described previously [12]. Primary antibodies employed in this study included: anti-β-catenin (1:1000, BD transduction Laboratories, USA), anti-Non-phospho (Active) β-catenin (Ser33/37/Thr41)(1:1000, Cell signaling Technology ), anti-cyclin D1 (1:1000, ABclonal), anti-*c-myc* (1:1000, ABclonal), anti-PARP (1:1000, Cell Signaling Technology), anti-caspase-3 (1:1000, Cell Signaling Technology), anti-cleaved caspase-3 (1:1000, Cell Signaling Technology), anti-GPX-4 (1:1000, ABclonal) and anti-actin (1:2000, Sigma-Aldrich). The membranes were then incubated with HRP anti-mouse IgG (Santa Cruz Biotechnology, USA) or anti-rabbit IgG (Santa Cruz Biotechnology) for 1h. Finally, the membranes were washed with TBS-T solution thrice for 10 minutes each. The Chemidoc MP imaging system (Bio-Rad, USA) and Image J software (NIH, USA) were used to visualize and quantify protein bands.

### Semi-Quantitative RT-PCR

Trizol reagent (Invitrogen, USA) was utilized to isolate total RNA following the manufacturer's instructions. cDNA synthesis, reverse transcription, and PCR were performed as previously mentioned [12]. Amplified DNA was separated using a 2% agarose gel through electrophoresis and was stained with ethidium bromide.

### Cell Viability Assay

SW480 and HCT116 cells (5000 cells/well) were seeded into 96-well plates and treated with TAGP for 48 h. A CellTiter-Glo assay kit (Promega) was utilized to evaluate the viability of each treated sample in triplicate following the manufacturer’s instructions.

### Intracellular Iron Assay

SW480 and HCT116 cells (5,000 cells/well) were incubated with TAGP for 48 h. The medium in the well was discarded and replaced with serum-free medium containing 1 μM FerroOrange (F374, Dojindo, Japan). The cells were then incubated for 30 min at 37°C. Finally, fluorescence was detected using a Zeiss Axiovert 200M fluorescence microscope (Carl Zeiss, Germany).

### Lipid Reactive Oxygen Species (ROS) Level Assay

Lipid ROS was identified using C11-BODIPY 581/591 (Thermo Fisher Scientific). Following a 48 h incubation, C11-BODIPY was exposed to cells in a medium at 37°C for 30 min. The fluorescence signals were then visualized with a fluorescence microscope.

### Apoptosis Analysis

An annexin V-FITC apoptosis detection kit (BioBud) was utilized to evaluated the apoptosis of each treated sample in triplicated according to the manufacturer’s instructions. Apoptotic cells were analyzed with a Cellometer Vision Image Cytometer (Nexcelom Bioscience, USA).

### Caspase-3/7 Assay

Caspase-3/7 activity was assessed using the Caspase-3/7 assay system from Promega. SW480 and HCT116 were seeded into a 96-well plate with a black bottom at 4,000 cells/well. After 48 h of treatment with TAGP, the caspase-3/7 reagent was added to each well and incubated at room temperature for 30 min. The luminescence signal was measured with a Victor 3 microplate reader.

### Statistical Analysis

Statistical comparisons of group means were made using the Student’s *t*-test. All experiments were performed in triplicate. Statistical significance was determined at *p* < 0.05 or *p* < 0.01. Findings are expressed as mean ± standard deviation (SD).

## Results

### TAGP Inhibits the Wnt/β-Catenin Pathway

**Fig. 1:**
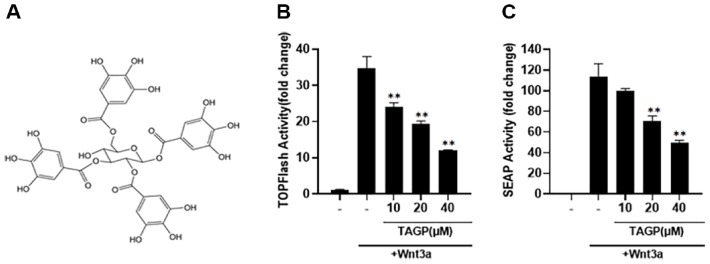
Identification of TAGP as an inhibitor of the Wnt/β-catenin pathway. (**A**) Chemical structure of TAGP. (**B, C**) In the presence of Wnt3a-CM, HEK293-FL and HEK293-SEAP reporter cells were incubated with either DMSO or the specified concentration of TAGP. After 15 h, FL (**B**) and SEAP (**C**) activities were measured. The results indicate the mean ± SD of three independent experiments. *, *p* < 0.05 and **, *p* < 0.01, compared with the Wnt3a- CM-treated control group.

To identify the bioactive compounds isolated from *Trapa japonica*, we employed TOPFlash reporter cells stably expressing a synthetic β-catenin-dependent firefly luciferase along with a human Frizzled-1 (hFz-1) overexpression in HEK293 cells. TAGP at various concentrations decreased luciferase activity in TOPFlash reporter cells, with activation occurring upon incubation in Wnt3a-CM (Fig. 1A and 1B). Additionally, TAGP demonstrated a dose-dependent reduction of Wnt3a-induced alkaline phosphatase activity in TOPAP reporter cells, containing the synthetic β-catenin-dependent secreted alkaline phosphatase (SEAP) along with hFz-1 overexpression (Fig. 1C). These findings indicate that TAGP specifically antagonizes the Wnt/ β-catenin pathway.

### TAGP Induces β-Catenin Phosphorylation and Proteasomal Degradation

**Fig. 2:**
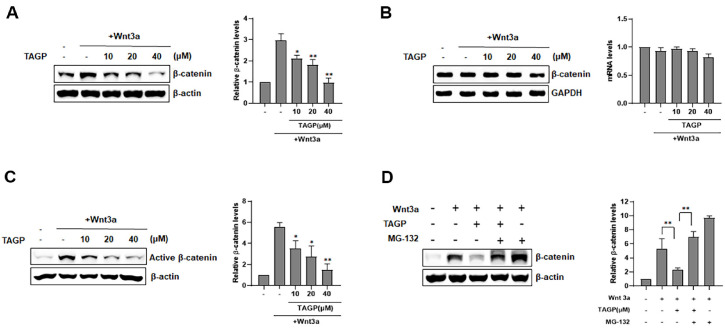
TAGP enhances the degradation of β-catenin via a proteasomal degradation pathway. (**A**) From HEK293-FL reporter cells treated with either DMSO or the indicated concentration of TAGP in the presence or absence of Wnt3a-CM for 15 h, cytosolic proteins were prepared and subsequently subjected. (**B**) Semi-quantitative RT-PCR was conducted to measure the levels of β-catenin and GAPDH using total RNA extracted from HEK293-FL reporter cells treated with either DMSO or the specified concentration of TAGP, with or without Wnt3a-CM, for 15 h. The levels of β-catenin mRNA were normalized to GAPDH levels. (**C**) Cytosolic proteins were prepared from HEK293-FL reporter cells treated with TAGP (10, 20, or 40 μM) in the presence of Wnt3a-CM for 15 h. Results of western blotting using an anti-non-phospho-β-catenin antibody. (**D**) HEK293-FL cells treated with TAGP were exposed to MG-132 (10 μM) for 8h. Cytosolic proteins were detected using an anti-β-catenin antibody. The levels of β-catenin and non-phosphorylated β-catenin were normalized to those of β- actin (**A, C**, and **D**). The results are expressed as mean ± SD of three independent experiments. *, *p* < 0.05 and **, *p* < 0.01 compared with the Wnt3a-treated vehicle control group.

The impact of TAGP on intracellular β-catenin level was assessed via western blot analysis. In line with the effects of TAGP. In line with its effects on β-catenin-responsive transcription (CRT), TAGP treatment showed a reduction of intracellular levels of β-catenin levels in TOPFlash cells exposed to Wnt3a-CM. However, treatment with TAGP at all tested concentrations did not influence the expression of β-catenin mRNA (Fig. 2B). Next, to clarify how TAGP facilitates β-catenin breakdown, we assessed whether TAGP facilitates the Ser33/37/Thr41 phosphorylation of β-catenin by western blot analysis with a non-phosphorylated β-catenin antibody. In the presence of Wnt3a-CM, the phosphorylation levels of β-catenin at the Ser33/37/Thr41 residues decreased, but increased with the addition of TAGP. (Fig. 2C). We then explored the role of the proteasome in TAGP-facilitated β-catenin turnover. The proteasome inhibitor MG-132 fully stopped the degradation of β-catenin that was caused by TAGP (Fig. 2D).

### TAGP Regulates β-Catenin Stability through a Pathway That Does Not Involve GSK-3β

**Fig. 3:**
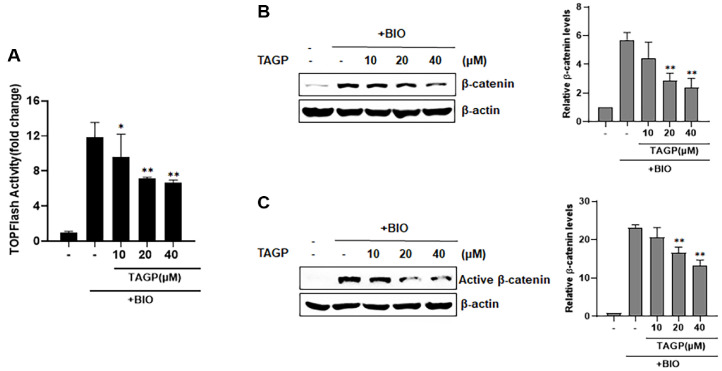
TAGP-mediated β-catenin phosphorylation and degradation through a GSK-3β independent mechanism. (**A**) HEK293-FL reporter cells were incubated with the specified concentrations of TAGP in the presence of 0.75 μM BIO. After 15 h, the FL activities were determined. (**B, C**) Cytosolic proteins were extracted from HEK293-FL reporter cells treated with TAGP (10, 20, or 40 μM) and 0.75 μM BIO for 15 h. Loading control was β-actin (**B, C**); the levels of β-catenin (**B**) and non-phosphorylated β-catenin (**C**) were normalized to that of β-actin. The results indicate the mean ± SD of three independent experiments. *, *p* < 0.05 and **, *p* < 0.01, compared with the BIO-treated control group.

In Wnt/β-catenin signaling, GSK-3β phosphorylates the Ser/Thr residues located at the N-terminus of β-catenin, which then bind to β-TrCP, becoming tagged for ubiquitination and subsequently broken down by the proteasome [13, 14]. In our experiment, we aimed to explore if GSK-3β contributes to the breakdown of β-catenin induced by TAGP, using the GSK-3β inhibitor BIO. To enhance CRT activity, we treated TOPFlash reporter cells with BIO. In Fig. 3A, we administered varying amounts of TAGP, leading to a reduction in CRT activity. Results from the western blot assay indicated that TAGP decreased the cytosolic β-catenin accumulation following BIO treatment (Fig. 3B). Moreover, we noted a decrease in the active β-catenin amount upon TAGP treatment (Fig. 3C). These findings indicate that TAGP lowers intracellular β-catenin levels via a pathway without involving GSK-3β.

### TAGP Reduces the Expression of β-Catenin-Responsive Genes in Colon Cancer Cells

**Fig. 4:**
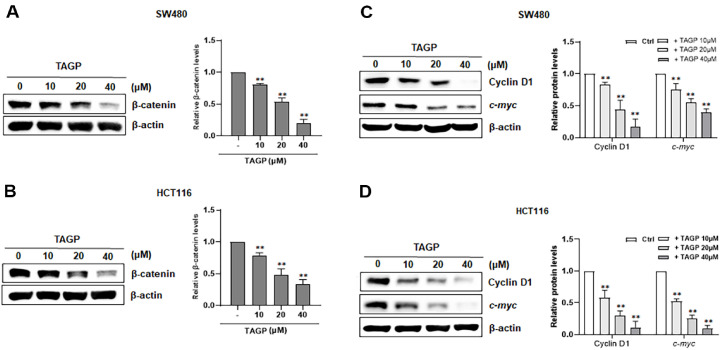
TAGP down-regulates the level of β-catenin and its target genes in colon cancer cells. (**A, B**) After treating SW480 and HCT116 cells with TAGP at 10, 20, or 40 μM concentrations for 15h, cytosolic proteins were isolated and analyzed by western blotting using an anti-β-catenin antibody. (**C, D**) Whole-cell extracts were obtained from SW480 and HCT116 cells after 24 h of incubation with TAGP. The extracts were analyzed using western blotting with anti-cyclin D1 and anti-*c-myc* antibodies. The results are presented as mean ± SD from three independent experiments. Statistical significance is indicated by *, *p* < 0.05 and **, *p* < 0.01, compared to the vehicle control group.

To measure the intracellular levels of β-catenin, colon cancer cells treated with TAGP were subjected to western blot analysis. Intracellular β-catenin levels in both SW480 and HCT116 cells were reduced upon treatment with TAGP (Fig. 4A and 4B). Next, we explored the influence of TAGP on the expression of of Cyclin D1 and c-Myc, which are regulated by β-catenin, in these cells. The treatment of colon cancer cells with various concentrations of TAGP led to the repression of Cyclin D1 and c-Myc level (Fig. 4C and 4D).

### Apoptosis Induction by TAGP Leads to the Inhibition of Colon Cancer Cell Proliferation

**Fig. 5:**
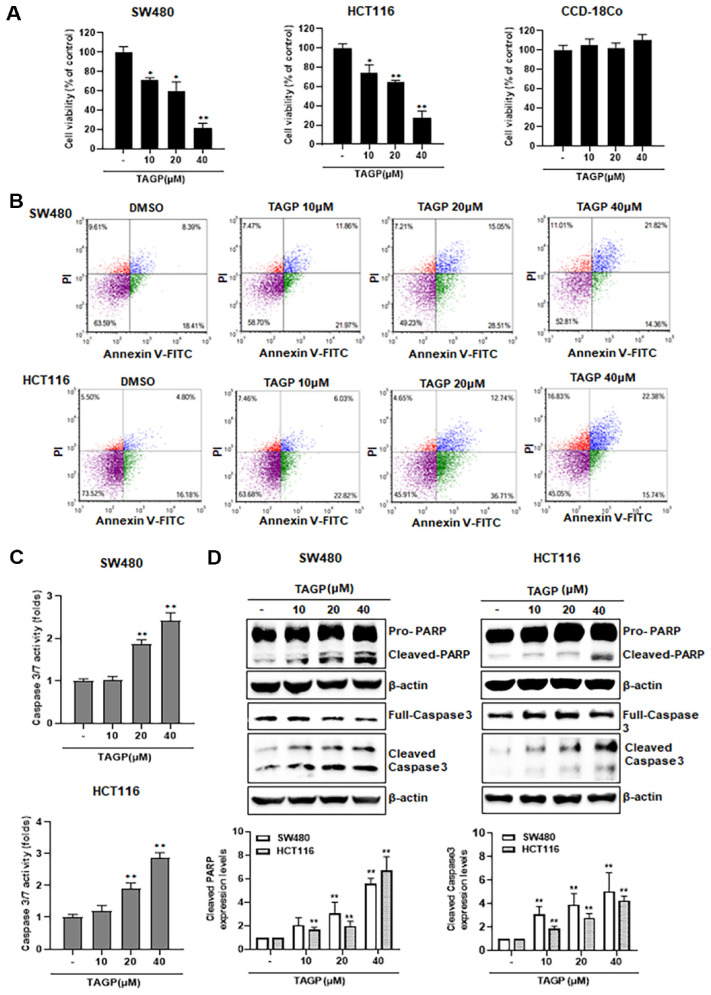
The effect of TAGP on colon cancer cell growth. (**A**) SW480, HCT116 and CCD-18Co cells were treated with different concentrations of TAGP (10, 20, or 40 μM) for 48 h. The CellTiter-Glo assay was employed for this experiment. (**B**) TAGP was administered to SW480 and HCT116 cells at concentrations of 10, 20, or 40 μM for 48 h. After incubation, Annexin V-FITC and propidium iodide (PI) were used to stain the cells. Annexin V-FITC and PI fluorescence intensity was represented on the x- and y-axes, respectively. (**C**) After treatment with TAGP, caspase-3/7 activity was assessed. Colon cancer cells were treated with the specified concentrations of TAGP for 48 h, and the FL activity was then measured. (**D**) For 48 h, SW480 and HCT116 cells were exposed to various concentrations of TAGP (10, 20, or 40 μM). Afterward, whole-cell extracts were analyzed for caspase-3, cleaved caspase-3, and poly (ADP-ribose) polymerase (PARP) using specific antibodies in a western blot analysis. The blots were re-probed with anti-actin antibodies as a control, and the results are presented as mean ± SD from three independent experiments. Statistical significance is indicated by *, *p* < 0.05 and **, *p* < 0.01, compared to the vehicle control group.

To examine the anti-proliferation effects of TAGP, we assessed the viability of colon cancer cells at different concentrations of TAGP. TAGP exhibited a concentration-dependent reduction in the viability of SW480 and HCT116 cell (Fig. 5A). However, TAGP did not affect the proliferation of CCD-18Co cells, which represent a line of normal human colon cells (Fig. 5A). To explore how TAGP may exert its antiproliferative effects, cytometry was conducted with Annexin V-FITC and propidium iodide (PI) staining to assess apoptosis. After TAGP treatment, the number of double-positive cells for annexin V-FITC and PI in SW480 and HCT116 cells rose in a manner that depends on the concentration (Fig. 5B). Furthermore, TAGP activated caspase-3 and -7 activities in SW480 and HCT116 cells, indicating the execution of apoptosis (Fig. 5C). Consistently, TAGP caused the proteolytic activation of pro-caspase-3 into its active form. Caspase-3 subsequently cleaved poly (ADP-ribose) polymerase (PARP), an important indicator of apoptosis (Fig. 5D). Our observations imply that TAGP induces apoptosis, leading to cytotoxic effects against colon cancer cells.

### TAGP Facilitates Ferroptosis in SW480 and HCT116 Cells

**Fig. 6:**
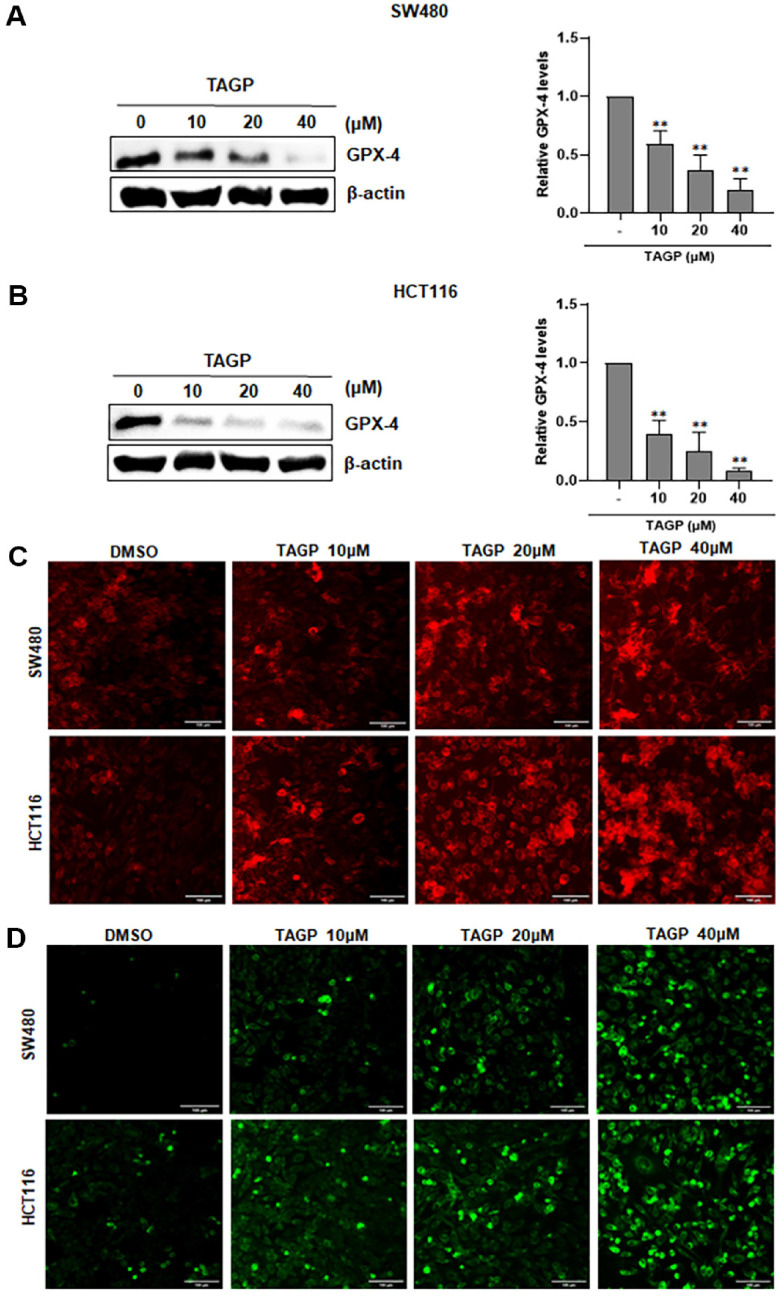
TAGP induces ferroptosis in SW480 and HCT116 cells. (**A, B**) Colon cancer cells were treated with either DMSO as a vehicle control or varying concentrations of TAGP for 48 h. Western blotting using anti-GPX-4 antibodies was conducted on whole-cell extracts. The results are presented as mean ± SD from three independent experiments. Statistical significance is indicated by *, *p* < 0.05, and **, *p* < 0.01, compared to the vehicle control group. (**C**) After 48 h of TAGP treatment, Fe^2+^ fluorescence in HCT116 and SW480 cells was observed using a FerroOrange probe. (**D**) Lipid peroxidation in SW480 and HCT116 cells was observed through fluorescence imaging using a C11-BODIPY-581/591 probe.

Ferroptosis represents a novel type of regulated cell death driven by oxidative stress and the iron-dependent peroxidation of lipids [25]. To investigate whether TAGP induces colon cancer cell death through ferroptosis, we examined the expression of GPX4, an essential regulator of ferroptosis, in SW480 and HCT116 cells using western blot analysis. The level of GPX-4 decreased with the addition of TAGP (Fig. 6A and 6B). Next, we utilized FerroOrange to measure the intracellular Fe2+ concentration. The results indicated a notable increase in the red fluorescence signal after TAGP treatment (Fig. 6C). We also used the C11-BODIPY 581/591 probe to detect lipid peroxidation and further examine lipid ROS accumulation. In line with our prediction, the accumulation of lipid ROS rose with increasing concentrations of TAGP treatment (Fig. 6D).

## Discussion

Colorectal cancer (CRC) holds the third highest rate of diagnosis among all cancers globally, according to WHO CLOBOVAN data from 2020. Present treatments for this cancer rely on surgical procedures and traditional cytotoxic medications, including 5-fluorouracil and oxaliplatin; nevertheless, the occurrence of side effects and drug resistance is frequently unavoidable. Consequently, there is an ongoing necessity to discover new therapeutics for CRC alongside the established cellular signaling pathways. In this respect, inhibiting Wnt/β-catenin signaling holds promise as a therapeutic intervention in CRC, as more than 94% of CRC cases exhibit mutations in at least one protein of this pathway, primarily the APC and FZD10, causing the pathway to be constitutively active [15]. This study is, to the best of our knowledge, the first to reveal the inhibitory effect of TAGP on the Wnt/β-catenin signaling pathway.

As a key mediator of Wnt/β-catenin signaling, β-catenin plays a crucial role in regulating cell growth, differentiation, and various developmental processes. The levels of β-catenin within the cell are primarily controlled by two APC-dependent pathways. In the APC/GSK-3β pathway, casein kinase 1 and GSK-3β collaborate to phosphorylate β-catenin in the presence of APC and Axin. This phosphorylated form is targeted by the E3 ubiquitin ligase β-TrCP, which facilitate its ubiquitination and subsequent degradation [13, 16]. Meanwhile, as Siah-1 binds to the APC, a ubiquitination complex is recruited that facilitates the turnover of β-catenin in the APC/Siah pathway [17]. This research provides multiple pieces of evidence suggesting that a novel degradation pathway, distinct from APC-dependent pathways, may participate in TAGP-mediated β-catenin destabilization. TAGP promoted the transformation of non-phosphorylated β-catenin into its phosphorylated version and facilitated the breakdown of β-catenin while a GSK-3β inhibitor was present. This indicates that TAGP promotes β-catenin degradation independently of GSK-3β. Notably, TAGP can still induce β-catenin degradation in colon cancer cells harboring APC mutations, indicating that APC does not participate in this process. Studies have shown that the retinoid X receptor (RXR) agonist enhances β-catenin degradation through a pathway that does not involve GSK-3β or APC [18]. Cyclin-dependent kinase 2 (CDK2)/cyclin A and protein kinase Cα (PKCα) promote β-catenin destabilization via a pathway that operates independently of the β-catenin destruction complex [19, 20]. Therefore, the increasing β-catenin turnover mediated by TAGP may involve PKCα and CDK2. Additional investigation is needed to reveal the mechanism by which TAGP mediates the breakdown of β-catenin.

Both apoptosis and ferroptosis were influenced by the Wnt/ β-catenin pathway via the promotion of survivin and glutathione peroxidase 4 (GPX4) expression, respectively [21, 22]. Apoptosis is a crucial programmed cell death process necessary for eliminating defective or tumorigenic cells. Of note, the intrinsic pathway, defined by the activation of caspase-3, has been shown to participate in drug-induced apoptosis [23]. In addition, ferroptosis refers to a form of regulated cell death driven by iron resulting from lipid peroxide accumulation in the cell membrane [24]. It is a crucial mechanism in regulating cancer cell proliferation, highlighting its potential as a therapeutic target in cancer [25]. This study clarified the growth-inhibitory mechanisms of TAGP on colon cancer cells by inducing ferroptosis and apoptosis. Specifically, TAGP triggered apoptosis by converting procaspase-3 to caspase-3 and ferroptosis by elevating intracellular lipid ROS and Fe^2+^ levels, while modulating the key ferroptosis-related protein GPX-4 in SW480 and HCT116 cells.

In summary, our chemical genetic strategy elucidated how TAGP exerts its anti-proliferative effects on colon cancer cells. TAGP induced both apoptosis and ferroptosis by accelerating the turnover of oncogenic β-catenin. Therefore, TAGP shows promise as an effective agent for cancer prevention in treating colorectal cancer. However, certain limitations of this study specifically focus on clarifying the processes driving the anti-cancer effects of TAGP *in vitro*. Consequently, future research should verify whether TAGP demonstrates the same mechanisms of action against β-catenin-driven tumorigenesis *in vivo*.
